# Profiling Microglia From Alzheimer’s Disease Donors and Non-demented Elderly in Acute Human Postmortem Cortical Tissue

**DOI:** 10.3389/fnmol.2020.00134

**Published:** 2020-10-28

**Authors:** Astrid M. Alsema, Qiong Jiang, Laura Kracht, Emma Gerrits, Marissa L. Dubbelaar, Anneke Miedema, Nieske Brouwer, Elly M. Hol, Jinte Middeldorp, Roland van Dijk, Maya Woodbury, Astrid Wachter, Simon Xi, Thomas Möller, Knut P. Biber, Susanne M. Kooistra, Erik W. G. M. Boddeke, Bart J. L. Eggen

**Affiliations:** ^1^Department of Biomedical Sciences of Cells and Systems, Section Molecular Neurobiology, University of Groningen, University Medical Center Groningen, Groningen, Netherlands; ^2^Department of Translational Neuroscience, UMC Utrecht Brain Centre, University Medical Centre Utrecht, University Utrecht, Utrecht, Netherlands; ^3^Foundational Neuroscience Center, AbbVie Inc., Cambridge, MA, United States; ^4^Neuroscience Discovery, AbbVie Deutschland GmbH and Co. KG, Ludwigshafen, Germany; ^5^Department of Cellular and Molecular Medicine, Center for Healthy Ageing, University of Copenhagen, Copenhagen, Denmark

**Keywords:** Alzheimer’s disease, microglia, single-cell RNA sequencing, barcoded Smart-seq2, human

## Abstract

Microglia are the tissue-resident macrophages of the central nervous system (CNS). Recent studies based on bulk and single-cell RNA sequencing in mice indicate high relevance of microglia with respect to risk genes and neuro-inflammation in Alzheimer’s disease (AD). Here, we investigated microglia transcriptomes at bulk and single-cell levels in non-demented elderly and AD donors using acute human postmortem cortical brain samples. We identified seven human microglial subpopulations with heterogeneity in gene expression. Notably, gene expression profiles and subcluster composition of microglia did not differ between AD donors and non-demented elderly in bulk RNA sequencing nor in single-cell sequencing.

## Introduction

Alzheimer’s disease (AD), one of the most prevalent age-related neurodegenerative disorders, is characterized by extracellular deposition of β-amyloid protein (Aβ) and intraneuronal neurofibrillary tangles in the neocortex (Hyman and Trojanowski, 1997).

Functional changes occurring in microglia cells have been proposed as an important factor in AD pathology (Zhang et al., 2013; Mhatre et al., 2015). AD single nucleotide polymorphism heritability was recently found to be highly enriched in microglia enhancers (Nott et al., 2019). Multiple genes associated with increased susceptibility for sporadic AD are preferentially expressed in microglia, including *APOE*, *CR1*, *CD33*, *INPP5D*, *PLCG2*, *MS4A6A*, and *TREM2* (Ulrich et al., 2014; Sarlus and Heneka, 2017). In AD mouse models, microglia have been implicated in Aβ seeding, Aβ plaques are surrounded by activated microglia, microglia protrusions physically interact with insoluble Aβ aggregates, and microglia around Aβ plaques undergo transcriptional changes (Rogers and Lue, 2001; Kamphuis et al., 2016; Keren-Shaul et al., 2017; Krasemann et al., 2017; Venegas et al., 2017; Yin et al., 2017). Sustained depletion of microglia in 5xFAD mice prevents Aβ plaque formation in parenchymal tissue and rather shows Aβ accumulation in the brain vasculature (Spangenberg et al., 2019). The functional changes occurring in microglia during AD pathology seem to be diverse (Friedman et al., 2018), and the exact role that microglia play in AD pathology is still unknown.

Many efforts have been made in AD mouse models to identify subpopulations of microglia that are associated with AD pathology. A subpopulation of microglia associated with neurodegeneration was discovered by Krasemann et al. (2017) that was associated with Aβ plaques and triggered by the phagocytosis of apoptotic neurons. This transcriptional phenotype was characterized by increased *Spp1*, *Itgax*, *Axl*, *Lilrb4*, *Clec7a*, *Ccl2*, *Csf1*, and *Apoe* and decreased *P2ry12*, *Tmem119*, *Olfml3*, *Csf1r*, *Rhob*, *Cx3cr1*, *Tgfb1*, *Mef2a*, *Mafb*, and *Sall1* expression levels (Krasemann et al., 2017). At the same time, a highly similar gene expression change, associated with microglia surrounding Aβ plaques was reported by Keren-Shaul et al. (2017), termed disease-associated microglia (DAMs). Using single-cell RNA-sequencing (scRNAseq) these DAMs were subdivided into two sequential stages, a *Trem2*-independent stage, marked by increased expression of *Tyrobp*, *Apoe*, and *B2m* and decreased expression of homeostatic genes, followed by a *Trem2*-dependent activation stage marked by induction of genes involved in lipid metabolism and phagocytosis (*Trem2, Spp1, Itgax, Axl, Lilrb4, Clec7a, Cts7, Ctsl, Lpl, Cd9, Csf1, Ccl6, Cd68*, and more). Sala Frigerio et al. (2019) described a microglia subpopulation in AppNL-G-F mice that appears in response to Aβ deposition and shares gene expression changes with DAMs. They identified mutually exclusive subtypes of activated response microglia overlapping with DAMs and, in addition, an independent subtype of interferon response microglia.

Studies investigating human microglia subtypes are limited, probably due to the technical and logistical difficulties of isolating pure, viable microglia from acute human brain tissue. Olah et al. (2018) investigated acutely isolated single human microglia from donors with a large variety of neuropathological backgrounds. They observed 23 clusters of microglia, where 5 out of 23 clusters were enriched for DAM signature genes. However, the neuropathological background of donors was too diverse to associate the observed changes with AD. Mathys et al. (2019) used single-nucleus sequencing and subclustered ~2,400 microglia of 48 donors. The study was focused on cell-type specific responses to AD development, and profiling of ~50 microglia per donor was insufficient to fully define microglia diversity in AD.

In this study, we aimed to identify transcriptomic changes in human microglia at the end stage of AD by applying both bulk and scRNAseq of microglia acutely isolated from postmortem central nervous system (CNS) tissue. We isolated and sequenced a pure population of microglia after CD11B+CD45+-based FACS sorting and investigated effects of sex, brain region, and diagnosis.

## Materials and Methods

### Human Brain Specimens

Autopsy brain specimens from the superior parietal lobe (LPS) and the superior frontal gyrus (GFS) were obtained from 25 donors of the Netherlands Brain Bank (NBB)^1^ and two donors of the NeuroBiobank of the Institute Born-Bunge (NBB-IBB, Wilrijk, Antwerp, Belgium, ID: BB190113). All donors have given informed consent for autopsy and use of their brain tissue for research purposes. The performed procedures and research protocols were approved by the corresponding ethical committees of the NBB. On average, the autopsies were performed within 6 h after death. Detailed information about brain specimens used for bulk and scRNAseq can be found in Supplementary Tables S1, S2, respectively.

### Microglia Isolation and Sorting

Microglia were isolated as described previously (Galatro et al., 2017a,b) with minor modifications. All procedures were performed on ice and all centrifugation steps were performed at 4°C. The tissue was homogenized by mechanical dissociation using a glass Dounce homogenizer in Medium A (HBSS (Gibco, 14170-088) containing 15 mM HEPES (Lonza, BE17-737E) and 0.6% glucose (Sigma–Aldrich, G8769) and was filtered through a 300- and 106-μm sieve. Homogenate was centrifuged at 220× *g* for 10 min, and myelin and other lipids were removed through two Percoll gradient centrifugation steps. A 100% Percoll solution was prepared consisting of 90% Percoll (GE Healthcare, UK) and 10% 10× HBSS (Gibco, 14180-046), from which the dilutions were prepared. First, cells were resuspended in 24.5% (vol/vol) Percoll in Medium A. A layer of PBS was added, and the gradient was centrifuged at 950× *g* for 20 min with reduced acceleration speed and brakes off. After the supernatant was removed, cells were resuspended in 60% (vol/vol) Percoll in 1× HBSS (Gibco, 14170-088), and a layer of 30% (vol/vol) Percoll in 1× HBSS (Gibco,) and PBS, respectively, were added and centrifuged at 800× *g* for 25 min (acc: 4, brake: 0). The cells in between the 30%/60% Percoll layer were collected and washed in 1× HBSS (Gibco, 14175-053) and pelleted at 600× *g* for 10 min.

Cells were incubated with antihuman Fc receptor (0.005 μg/ml eBioscience, 14-9161-73) for 10 min in Medium A without phenol red (HBSS, Gibco, 14170-053) containing 15 mM HEPES (Lonza, BE17-737E), 0.6% glucose (Sigma–Adrich, St. Louis, MO, USA, G8769), 1 mM EDTA (Invitrogen, 15575-038), followed by incubation with FITC antihuman CD45 (5 μg/ml, BioLegend, 304006) and PE antihuman CD11B (3.75 μg/ml, BioLegend, 301306). Prior to sorting, DAPI (0.15 μg/ml, Biolegend, 422801) and eBioscience DRAQ5 (2 μM, Thermo Fisher Scientific, Waltham, MA, USA, 63351) were added. Single, viable microglia defined as DAPI−, DRAQ5+, CD45+, and CD11B+ were FACS sorted on a Beckman Coulter MoFlo XDP or Astrios. Microglial subpopulations might be reflected by a slight difference in CD45 and CD11B expression. Since only scRNAseq allows for the disentanglement of microglial subpopulations, we applied a broader CD45+CD11B+ gate to collect microglia for scRNAseq and a narrower CD45+CD11B+ gate for bulk RNA sequencing (bulk RNAseq).

For bulk microglia RNAseq, microglia were sorted into low-retention Eppendorf tubes (Sigma–Adrich, St. Louis, MO, USA, Z666548-250EA) containing 200 μl RNA later (Qiagen, 76104). Following centrifugation at 4°C and 5,000× *g* for 10 min, supernatant was carefully removed, and microglia were resuspended in 350 μl RLTplus lysis buffer (Qiagen, 1053393) and stored at −80°C. For barcoded 3’ single-cell sequencing, 15,792 single microglia were collected in 384-well PCR plates containing cell lysis buffer [0.2% Triton (Sigma–Adrich, St. Louis, MO, USA, T9284), 4 U RNAse inhibitor (Takara, 2313A), 10 mM dNTPs (Thermo Fisher Scientific, Waltham, MA, USA, #R0193), and 10 μM barcoded oligo-dT primer] and were stored for maximally 1 month at −80°C until further processing. For 10× Genomics Chromium single-cell RNA sequencing, approximately 25,000 single microglia were sorted from each sample (2018-135, 2019-010) into low-retention Eppendorf tubes (Sigma, Z666548-250EA) containing 5 μl Medium A and were immediately processed with the Single Cell 3’ Reagent Kit v2 (10× Genomics). FACS data was analyzed with FlowJo (Becton, Dickinson and Company).

### Bulk Microglia RNA Sequencing Library Preparation

Total RNA was extracted from the bulk sorted microglia samples using the RNeasy Plus Micro kit (Qiagen, 74034) according to the manufacturer’s protocol. RNA quality and quantity were determined with the Experion RNA HighSens Analysis Kit (Bio-Rad, #7007105). All 25 RNA samples, with RIN values varying between 5.1 and 9.9, were enriched for poly(A) + messenger RNA using NEXTflex Poly(A) Beads (BIOO Scientific, #NOVA-512980) according to the manufacturer’s protocol, and 14 μl of this mRNA-enriched poly(A)-tailed RNA was used as the input for the NEXTflex Rapid Directional qRNA-Seq kit (BIOO Scientific, #NOVA-5130-04). Library preparation was performed according to the manufacturer’s protocol. Quality and concentration of libraries from individual samples were assessed using the High Sensitivity dsDNA kit (Agilent, 067-4626) on a 2100 Bioanalyzer (Agilent) and a Qubit 2.0 Fluorometer (Life Technologies). Subsequently, individual libraries were combined into two sequencing pools of 13 samples each with equal molar input, and 75 bp paired-end sequencing was performed on an Illumina NextSeq 500 system. PhiX was added at 5% to both pools as an internal control before sequencing.

### Single-Cell RNA Sequencing Library Preparation

The scRNAseq library preparation method that was used here is based on the Smart-seq2 protocol by Picelli et al. (2014) with the modification of obtaining 3’ instead of full-length RNA/cDNA libraries as in Uniken Venema et al. (2019). After cell lysis and barcoded poly-dT primer annealing (73°C, 3 min), RNA was reversed transcribed (RT) based on the template switching oligo mechanism using 0.1 μM biotinylated barcoded template switching oligo (BC-TSO, 5’-AAGCAGTGGTATCAACGCAGAGTACATrGrG+G-biotin-3’), 25 U SmartScribe reverse transcriptase, first-strand buffer, and 2 mM DTT (Takara, 639538), 1 U RNAse inhibitor (Takara, 2313A), and 1 M betaine (Sigma–Aldrich, B0300-5VL) with the following PCR program: (1) 42°C, 90 min; (2) 11 cycles of 50°C, 2 min, 42°C, 2 min; (3) 70°C, 15 min. To account for amplification bias and to allow multiplexing of cells, the barcoded poly-dT primer contains a cell-specific barcode and a unique molecular identifier (UMI) and a known sequence that is used as a primer-binding site during the first amplification step. This same primer-binding site is linked to the BC-TSO, enabling the use of one primer pair (custom primer) during the first amplification. After the RT reaction, primer-dimers and small fragments were removed by 0.5 U Exonuclease (GE Healthcare, E70073Z) treatment for 1 h at 42°C. cDNA libraries were amplified with KAPA Hifi HotStart ReadyMix (KAPA Biosystems, KK2602) and custom PCR primer (5′-AAGCAGTGGTATCAACGCAGAGT-3′) with the following PCR program: 98°C, 3 min, 25 cycles of 98°C, 20 s, 67°C, 15 s, 72°C, 6 min; 72°C, 5 min. cDNA libraries of 84 cells were multiplexed, and short fragments were eliminated by Agencourt Ampure XP beads (Beckman Coulter, A63880, ratio of 0.8:1 beads to library volume). The quality of multiplexed cDNA libraries was examined with a 2100 Bioanalyzer (Agilent) according to the manufacturer’s protocol. cDNA libraries with an average size of 1.5–2 kb were tagmented and indexed during a second PCR amplification step with the Illumina Nextera XT DNA preparation kit (Illumina, FC-131-1024). Tagmentation was performed according to the manufacturer’s protocol with an input of 500 pg cDNA and amplicon tagment mix for 5 min at 55°C. The tagmentation reaction was stopped using neutralize tagment buffer. Next, tagmented cDNA was amplified with Nextera PCR master mix, the Nextera indices (12 pool-specific indices, Illumina, FC-131-2001) and 10 μM P5-TSO hybrid primer (5′-AATGATACGGCGACCACCGAGATCTACACGCCTGTCCGCGGAAGCAGTGGTATCAACGCAGAGT*A*C-3′) with the following PCR program: (1) 72°C, 3 min; (2) 95°C, 30 s; (3) 10 cycles of 95°C, 10 s, 55°C, 30 s, 72°C, 30 s; and (4) 72°C, 5 min. Tagmented cDNA libraries were purified by a 0.6:1 ratio of Agencourt Ampure XP beads (Beckman Coulter, A63880) to library volume. The quality and concentration of tagmented cDNA libraries were determined with a 2100 Bioanalyzer (Agilent). cDNA pools with an average size of 300–600 bp were multiplexed using a balanced design with pools from 10 different donors (in total, 840 cells) per sequencing run. In other words, cells from each donor were distributed over several sequencing runs to avoid potential batch effects. To eliminate short fragments, the final superpool was gel-purified from 2% agarose gel (Invitrogen, 10135444) with the Zymoclean Gel DNA Recovery kit (Zymo Research, D4007). The concentration was determined using a 2100 Bioanalyzer (Agilent) and Qubit 3.0 (ThermoFisher Scientific) according to the manufacturer’s protocol. Pools were loaded on an Ilumina NextSeq at a final concentration of 2 pM with a 7% spike in PhiX DNA; 0.3 μM BC read 1 primer (5′-GCCTGTCCGCGGAAGCAGTGGTATCAACGCAGAGTAC-3′) was used for the sequencing run. The libraries were sequenced on an Illumina NextSeq 500 system with an average sequencing depth of 26 million reads per pool. The exact number of cell barcodes per pool varied, but approximately ~350,000 raw reads per cell were sequenced. After read alignment, exonic read count, and deduplication, this resulted in an average of 19,050 UMIs per cell.

### 10× Genomics Chromium Single-Cell 3’ Library Construction

The scRNAseq barcoded libraries were constructed according to the instructions of the Single-Cell 3’ Reagent Kits v2 (10× Genomics). Briefly, cells were loaded into a slot of a Chromium chip and GEMs were incubated in a thermal cycler to generate barcoded cDNA. After amplification, the cDNA was fragmented and processed for sequencing by ligating adapters and sample indices. The libraries were sequenced on an Illumina NextSeq 500 system with an average sequencing depth of ~42,500 raw reads per cell; this resulted in an average of 629 UMIs per cell.

### Immunohistochemistry

Immunohistochemistry was performed as described previously (Yin et al., 2017). Briefly, 16 μm sections of PFA-fixed human brain tissue were vacuum-dried, post-fixated for 10 min with 4% PFA, and washed with PBS. Heat-induced antigen retrieval was performed in sodium citrate solution (pH 6.0) for 10 min in a microwave. Endogenous peroxidase was blocked by incubating the slides in 0.3% H_2_O_2_ for 30 min. After three washing steps with PBS, primary antibodies against IBA1 (WAKO, 019-19741, 1:1,000), Phospho-TAU (Thermo Fisher Scientific, Waltham, MA, USA, MN1020, clone AT8, 1:750), and Beta-Amyloid (Dako, M0872, 1:100) were diluted in Bright Diluent (ImmunoLogic, BD09-500) to prevent background staining and incubated overnight at 4°C. After three washing steps in PBS, secondary biotinylated horse antimouse IgG antibody (0.000125 mg/ml Vector BA-2001) was incubated for 1 h at room temperature. The tissue sections were washed three times in PBS. The signal was amplified with avidin-biotin complexes (Vectastain Elite ABC-HRP, Vector, PK-6100) for 30 min at RT and visualized with 3,3′-diaminobenzidine (Sigma–Adrich, St. Louis, MO, USA, D-5637). Additionally, after the phospho-TAU staining, we performed a crystal violet counterstaining. The slides were dehydrated with an ethanol series (50%, 70%, 80%, 90%, 2× 96%, and 3× 100% ethanol) and air-dried for 30 min prior to mounting a coverslip with DePeX (Serva, 18243). Imaging was performed with a Hamamatsu Nanozoomer at 40x magnification.

### Preprocessing of RNA-Sequencing Data

For bulk samples, NEXTflex barcodes (nine base pairs) were stripped from the sequence. Sequencing reads were then aligned with HISAT2 (version 2.1.0; Kim et al., 2015) to the GRCh38.92 reference genome with Ensembl annotation. Further processing with samtools (version 1.9) and Picard Tools (version 1.140) included sorting, read group assignment, verification of mate pair information, and marking of duplicates. Reads were further quantified using featureCounts (Subread version 1.6.2; Liao et al., 2014) and based on NEXTflex barcodes deduplicated with a bash script developed by BIOO Scientific (version 2, release date 11/1/14) to eliminate PCR duplicates.

Reads from bc-Smart-seq2 single cells were demultiplexed with UMI-tools (version 0.5.3; Smith et al., 2017). A cell barcode whitelist was used to filter barcodes for downstream processing. Cell barcodes and the UMI from each read were extracted to the read name of the sequence using the UM-tools *extract* function. Reads were single-end aligned with HISAT2 (version 2.1.0) to the GRCh38.91 reference genome with Ensembl annotation using default parameters, followed by sorting and indexing of BAM files. Primary counts were quantified with featureCounts (version 1.6.0) using the flag -primary. PCR duplicates were removed, and unique molecules were counted per gene and per cell using the UMI-tools function *count* (Smith et al., 2017). Nine pools in which less than 10% of total sequenced reads were assigned to features were excluded.

Reads from 10× Genomics Chromium single cells were demultiplexed and aligned with Cell Ranger to the GRCh38 genome with Ensembl transcriptome annotation using default parameters. Barcode filtering was performed with the R package DropletUtils using a threshold of >100 UMIs per barcode (Griffiths et al., 2018).

### Downstream Analysis

**Figure 1:**
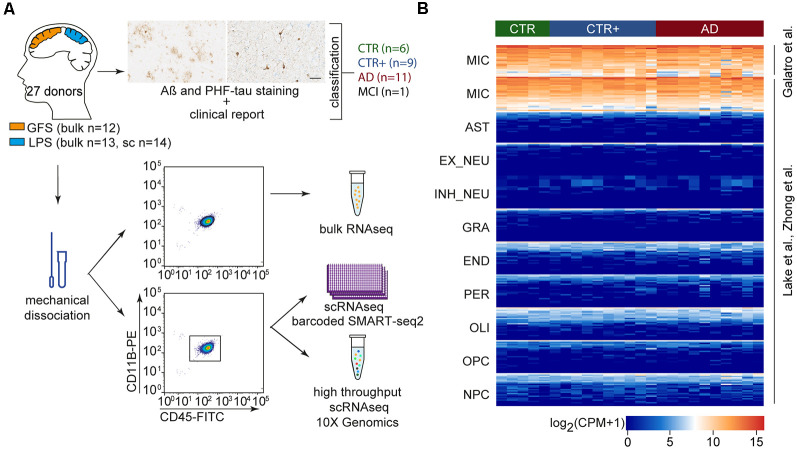
Microglia gene expression profiling of four groups of donors from acute postmortem human brain tissue. **(A)** Tissue samples from GFS and LPS were classified into four experimental groups (CTR, CTR+, AD, MCI) based on Aβ and PHF-tau immunohistological stainings and the clinical report of the donor (scale bar = 50 μm). Microglia were mechanically isolated and collected by CD11B+CD45+ -based FACS sorting. Sorted microglia were used for bulk or single-cell sequencing (barcoded Smartseq2 and 10× Genomics sequencing techniques). **(B)** Heat map depicting log_2_ CPM expression of cell type-specific markers in bulk-sorted microglia, as previously published in different data sets (Lake et al., 2016; Galatro et al., 2017a; Zhong et al., 2018). Abbreviations: AD, clinically diagnosed Alzheimer’s Disease; AST, astrocytes; Aβ, amyloid beta; CPM, counts per million; CTR, Control; CTR+, Control with Aβ plaques and/or hyperphosphorylated tau; END, endothelial cells; EX_NEU, excitatory neurons; GFS, superior frontal gyrus; GRA, granulocytes; IN_NEU, inhibitory neurons; LPS, superior parietal lobe; MCI, mild cognitive impairment; MIC, microglia; NPC, Neural Progenitor Cells; OLI, oligodendrocytes; OPC, Oligodendrocyte Precursor Cells; PER, pericytes; PHF-tau, hyperphosphorylated tau; scRNAseq, single-cell RNA sequencing.

Samples were sequenced with a median of 32 million total reads, 25 million uniquely mapped reads, and 20 million exonic reads. The sequencing depth fulfilled the ENCODE Consortium guidelines for RNA sequencing experiments with the aim to investigate the similarity between transcriptional profiles of polyA+ samples (The ENCODE Consortium, 2016). A data-adaptive flag method (George and Chang, 2014) was applied to remove lowly expressed genes. Only genes with an expression level higher than three counts per million (CPM) in at least two samples were included in the analysis. After both gene-filtering steps, the average library size was 3.8 M counts (standard deviation ±2.2 M) for downstream analysis. Counts per million (CPM) were calculated as (counts gene *i*/sample library size) × 10^6^. For plotting gene expression in boxplots and heat maps log_2_(CPM + 1) was used. For Figure 1B, cell type markers from three independent data sets (Lake et al., 2016; Galatro et al., 2017a; Zhong et al., 2018) were combined, and the top 25 most abundant genes were plotted. Principal component analysis was computed on rlog transformed counts of the top 500 most variable genes using the *prcomp* function. We applied upper quartile normalization to adjust for library size with *calcNormFactors* function of edgeR (version 3.28.1; Robinson et al., 2010). For the design matrix of the within-subject comparison of the brain region, we used the factors “Brain Region” and “Donor.” For the between-subject comparison between the sexes, we included “Sex” while controlling for “Age.” For the between-subject comparison of donor conditions, we used the factor “Donor Group” (CTR, CTR+, AD) while controlling for variables “Age” and “Sex.” The brain regions (LPS and GFS) were analyzed separately for between-subject comparisons. Differences between groups were tested with likelihood ratio tests as implemented in edgeR, resulting in FDR values. For a subset of significantly altered genes, we observed a large absolute increase in predictive log fold changes (logFC) computed by edgeR compared to regular log fold changes computed by subtracting average log_2_CPMs between groups. We opted to report the most conservative of the two, in this case the regular logFC. Thresholds were set at FDR<0.05 and abs(logFC)>1 to define differentially expressed genes.

For bc-Smart-seq2 single cells, approximately 25% of the cells were filtered out during preprocessing. To remove empty cells while respecting variation across donors, we set a threshold for each donor individually, removing cells with library sizes exceeding median of log(total counts) ±3 median absolute deviation (MAD). In addition, cells with more than 3,000 unique genes and were considered doublets (genes per cell median 520; MAD ± 276) and were excluded. Cells with >10% mitochondrial transcripts were excluded. Genes not detected in at least three cells were removed. Downstream analysis started with 14 donors and 9,764 cells for clustering analysis. After filtering, we detected a median of 13,441 UMIs and median 513 unique genes per cell. We clustered a median of 714 cells per donor for bc-Smart-seq2 data. Raw counts were normalized by total expression per cell, scaled by 10,000, and log-transformed with the CRAN package Seurat (version 3.1.5; Butler et al., 2018). We used the mean variability method to select highly variable genes (HVGs). Briefly, this method identifies variable genes while controlling for the strong relation between gene variability and gene average expression. We allowed lowly expressed genes in the highly variable gene list, since some disease-associated genes (e.g., *TREM2, TYROBP, CTSD*) are biologically relevant but also lowly expressed. These extra ~600 lowly expressed HVGs did not change clustering results and were included in the final clustering analysis. The number of detected genes, ribosomal, and mitochondrial content per cell were regressed out as they were considered unwanted sources of variation. In addition, smaller technical variations due to sequencing superpool or i7 sample index were regressed out. We used the first 15 principal components for PCA-Louvain clustering as implemented by Seurat (version 3.1.4). Cluster resolution was set at 0.5 since seven clusters was the most stable cluster number when considering resolutions from 0.1 to 2.0. Resolutions above 0.5 were investigated and did not give AD-associated subclusters. Cluster-enriched genes were identified using logistic regression as implemented in Seurat’s *FindAllMarkers* function with default thresholds and only.pos = TRUE. Gene ontology enrichment (GO) for cluster-enriched genes was computed against the human genome as background (“org.Hs.eg.db” version 3.10.0) using ClusterProfiler (version 3.14.3; Yu et al., 2012) with *p*-value cutoff of 0.01 and q-value cutoff of 0.05.

10× Genomics Chromium single cells were analyzed for each donor individually. Low-quality cells with >10% mitochondrial gene (MT) content were removed in donor 2018-135. Donor 2019-010 had very high cell quality, so a >5% MT threshold was applied. Duplicate cells were filtered by setting an upper UMI threshold that was based on the multiplet rate as mentioned in the 10× Genomics user guide. Genes not detected in at least three cells were removed. We analyzed 3079 single cells for MCI donor 2019-010 and 2,881 single cells for AD donor 2018-135. We regressed out total UMI count, ribosomal, and mitochondrial content per cell. The first 20 principal components were used for PCA-Louvain clustering. Each donor was analyzed individually. To gain sufficient detail to detect small subpopulations within one donor, the cluster resolution was set at 0.6 for each donor. Cluster-enriched genes were identified using logistic regression implemented in Seurat’s *FindAllMarkers* function with default thresholds and only.pos = TRUE.

### Gene Set Analysis

Raw counts were normalized by total expression per cell, scaled by 10,000, and log transformed. The DAM gene set used here consists of the top 500 most significantly increased mouse genes between *microglia3* and *microglia1* from Keren-Shaul et al. (2017) subsetting to genes expressed and detectable in human single microglia. The neurodegeneration-related gene set consists of the 126 human genes from Friedman et al. (2018), reported in Data S4 as myeloid activation “neurodegeneration-related.” For the single-nucleus gene set, 77 cluster marker genes from *microglia1* cluster reported by Mathys et al. (2019) were used. Gene set score was defined as average expression of genes in a set per cell. Next, the mean gene set score in any cluster was calculated and compared to the mean of all other clusters. To compare gene set cluster means we used linear regression with gene set score as dependent variable and independent variables cell library size (*z*-transformed), number of detected genes per cell (z-transformed), donor, and the cluster number as categorical variable. *P*-values were adjusted with a Bonferroni correction. Visualizations were made with the R package “ggplot2.”

## Results

### Isolation of Pure Microglia From Acute Postmortem Brain Tissue

To investigate transcriptomic changes in microglia during AD, bulk and single-cell RNA sequencing (scRNAseq) were performed. Postmortem tissue samples of the superior parietal lobe (LPS) and superior frontal gyrus (GFS) were obtained from 27 donors (Supplementary Tables S1, S2). The samples were classified into three experimental groups based on a clinical diagnostic report provided by the Netherlands Brain Bank/NeuroBiobank Born-Bunge and immunohistochemical analysis of Aβ and hyperphosphorylated tau (PHF-tau): CTR (no dementia, absence of Aβ plaques and PHF-tau, *n* = 6), CTR with plaques (CTR+, no dementia, presence of Aβ plaques and/or PHF-tau, *n* = 9), and AD (dementia, AD diagnosis, presence of Aβ plaques and/or PHF-tau, *n* = 11). One donor diagnosed with mild cognitive impairment (MCI) and presence of Aβ and PHF-tau plaques was included as well. Representative images of immunohistochemical Aβ and PHF-tau stainings of donor CNS tissues are shown in Supplementary Figure S1. The stratification of CTR and CTR+ donors ensured that the CTR group was free of undiagnosed AD donors. Microglia were isolated from mechanically dissociated tissue using fluorescence-activated cell sorting (FACS) of single, viable CD11B and CD45 positive cells. Twenty-five microglia samples (13 LPS and 12 GFS) from 17 donors were sequenced as bulk samples, and 14 LPS samples from 14 donors were single-cell sequenced (bc-Smart-seq2 and 10× Genomics; Figure 1A). Four donors (1 CTR, 3 CTR+) were included in both single-cell and bulk cohorts. A pure microglia population was obtained based on the expression of known microglia marker genes and the absence of expression of genes associated with other CNS cell types (Figure 1B). Cell type–specific genes were selected based on previously published gene expression profiles of adult human microglia (Galatro et al., 2017a), early human prefrontal cortex cell types (Zhong et al., 2018), and human CNS nuclei (Lake et al., 2016).

### No Differences in Bulk RNA Sequencing Profiles of Microglia Between CTR, CTR With Plaques (CTR+), and AD Donors

To investigate general transcriptional characteristics of microglia in AD, we bulk sorted and transcriptionally profiled microglia from CTR (*n* = 3), CTR+ (*n* = 7) and AD (*n* = 7) samples. The CTR, CTR+, and AD samples had comparable sequencing depth (Supplementary Figures S2A–C) and fulfilled the recommended sequencing depth of ENCODE Consortium guidelines. In addition, median RNA integrity numbers were ~8–9, indicating high RNA quality (Supplementary Figure S2D). Thus, bulk gene expression profiles of microglia were likely not influenced by quality metrics, such as sequencing depth and RIN value.

**Figure 2:**
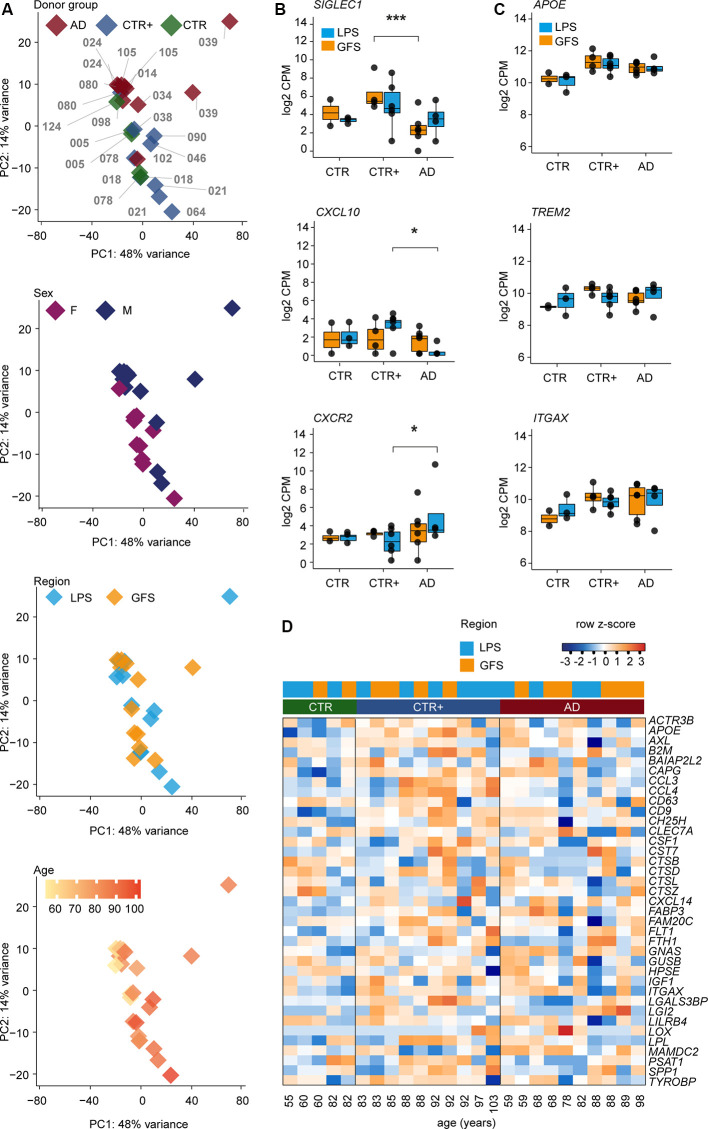
Transcriptomic analysis of microglia populations isolated from CTR, CTR+, and AD donors. **(A)** Principal component analysis (PCA) of RNA-sequencing data from acutely isolated microglia illustrating the effect of donor (each sample is indicated with a donor label), donor groups, sex, brain region, and age. **(B)** Differentially expressed genes between AD and CTR+ donors (likelihood ratio test, *SIGLEC1* ***FDR = 0.002, *CXCL10* *FDR = 0.02 and *CXCR2* *FDR = 0.02). **(C)** Selected examples of expression levels (log_2_ CPM) of disease-associated microglia genes in bulk microglia samples. **(D)** Heat map depicting expression of most abundant disease-associated microglia genes in bulk microglia samples from three donor groups in row z scores of log_2_ CPM values. Donors are ordered by age (young to old) within the donor group. Abbreviations: AD, Alzheimer’s Disease; CPM, counts per million; CTR, Control; CTR+, Control with Aβ plaques and/or hyperphosphorylated tau; F, female; M, male; GFS, superior frontal gyrus; LPS, superior parietal lobe.

Principal component analysis (PCA) revealed no clear segregation between donor groups (Figure 2A). Variation in the first principal component could potentially be attributed to individual gene expression differences between donors. The second principal component showed segregation of microglia samples on sex and age but not on brain region.

To further examine the effect of sex, male and female microglia samples from GFS (female *n* = 5, male *n* = 7) and LPS (female *n* = 6, male *n* = 7) were compared while accounting for the effect of age. Besides the expected expression differences of genes located on sex chromosomes, two genes in GFS and seven genes in LPS, which were localized on autosomal chromosomes, were differentially expressed in microglia from male compared to female donors. None of the differentially expressed genes located on autosomal chromosomes overlapped between the GFS and LPS (Supplementary Table S3). This suggests that, besides the expression of genes located on sex chromosomes, microglia gene expression profiles of males and females are similar.

Gene expression differences between microglia from frontal and parietal brain regions were assessed for eight donors, and a within-subject comparison was performed. Expression of four genes (*CST7, HBEGF*, *JAML*, *TREM1*) was increased in LPS compared to GFS, and one long noncoding RNA (*AC011451.1*) was decreased in LPS compared to GFS (Supplementary Figure S3, Supplementary Table S4). This indicates that the bulk gene expression profiles of microglia isolated from frontal (GFS) and parietal (LPS) brain regions are very similar in terms of gene expression.

To assess the effect of AD, we compared microglia from the AD group to the CTR/CTR+ group. The effect of age and sex in the AD-CTR/CTR+ comparison was accounted for, because subtle effects were visible in the PCA. LPS- and GFS-derived microglia were analyzed separately.

Microglia from the AD group were compared to the CTR group. In GFS microglia, the expression level of one gene was significantly increased (CTR *n* = 2, AD *n* = 6), but this was one finding using a small reference group (CTR *n* = 2). In LPS, no significant gene expression changes were detected (CTR *n* = 3, AD *n* = 4; Supplementary Table S5). Therefore, no gene expression differences were observed between the CTR and AD group.

Next, microglia from the AD group were compared to the CTR+ group. In GFS (CTR+ *n* = 4, AD *n* = 6), the expression level of four genes was increased, and 13 genes were decreased in AD. For example, expression of the phagocytic marker S*IGLEC1* (Bogie et al., 2018) was decreased in AD compared to CTR+ microglia (Figure 2B, Supplementary Table S5). In LPS (CTR+ *n* = 6, AD *n* = 4), five genes were less expressed in AD compared to CTR+ microglia, including *CXCL10* (Figure 2B, Supplementary Table S5). *CXCL10* has been implicated in AD studies with contrasting results. *CXCL10* protein levels were reported to be increased in AD donors in prefrontal cortical tissue (Bradburn et al., 2018). However, *CXCL10* protein levels were increased in MCI donors but not in severe AD donors in the cerebral spinal fluid (Galimberti et al., 2006). Expression of 20 genes was increased in the LPS of AD compared to CTR+ microglia, including chemokine receptors *CXCR1* and *-2* (Figure 2B, Supplementary Table S5). Several studies claim that *CXCR2* could augment AD pathology in mouse models and that deficiency in *CXCR2* decreases amyloid-beta deposition (Xia and Hyman, 2002; Bakshi et al., 2011; Veenstra and Ransohoff, 2012; Ryu et al., 2015). All gene expression differences detected between AD and CTR+ microglia were restricted to either the GFS or the LPS brain region (Supplementary Table S5).

In 5xFAD mice, a subset of microglia is reported that emerges with increasing amyloid pathology. These disease-associated microglia (DAMs) progressively express a specific set of genes, associated with lipid metabolism and phagocytosis (Keren-Shaul et al., 2017). Expression levels of the most significantly increased genes in the mouse DAM cluster, including *APOE*, *TREM2*, *ITGAX*, were investigated in our bulk human microglia data set and did not differ between control- and AD-derived human microglia in both LPS and GFS regions (Figures 2C,D, Supplementary Table S5).

To summarize, although some gene expression changes were detected in CTR+ compared to AD bulk microglia, microglia transcriptomes from AD and CTR donors did not significantly differ.

### Single-Cell Gene Expression Profiling Identifies Seven Subsets of Microglia but No AD-Associated Subpopulation

**Figure 3:**
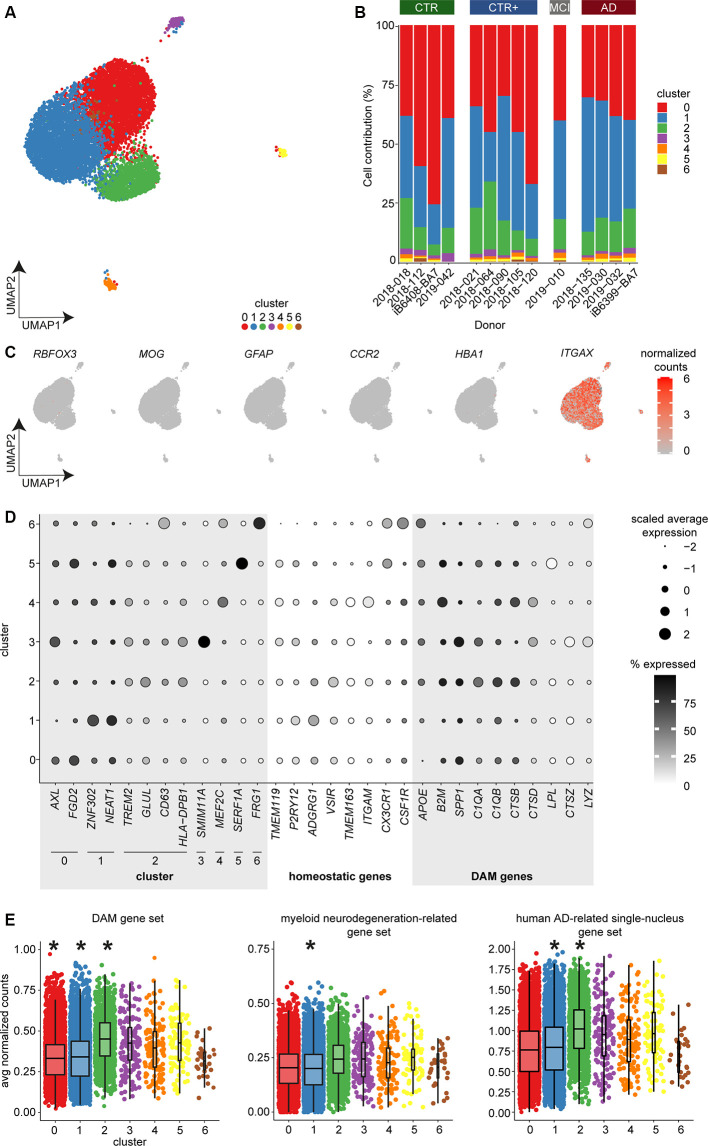
Single-cell expression profiling of microglia identified seven subsets of microglia but no AD-associated cluster using bc-Smart-seq2. **(A)** UMAP visualization and unsupervised clustering of microglia derived from AD (*n* = 4), MCI (*n* = 1), CTR+ (*n* = 5), and CTR (*n* = 4) donors. Colors represent cluster identity. **(B)** Cluster contribution normalized to donor. **(C)** Expression of cell type–specific markers for neurons (*RBFOX3*), oligodendrocytes (*MOG*), astrocytes (*GFAP*), circulating monocytes (*CCR2*), erythrocytes (*HBA1*), and microglia (*ITGAX*). **(D)** Dot plot visualizing scaled average expression and the percentage of cells expressing the indicated genes. **(E)** Average expression of the mouse DAM (Keren-Shaul et al., 2017), myeloid neurodegeneration-related (Friedman et al., 2018), and human AD-related single-nucleus (Mathys et al., 2019) gene sets per cell across clusters (multiple linear regression with Bonferroni correction, *indicate *p*-value < 0.001). Horizontal lines in the boxplots represent the mean; the lower and upper hinges represent the 25th and 75th percentiles. Abbreviations: AD, Alzheimer’s Disease; avg, average; CPM, counts per million; CTR, Control; CTR+, Control with Aβ plaques and/or hyperphosphorylated tau; DAM, disease-associated microglia; MCI, mild cognitive impairment; UMAP, uniform manifold approximation and projection.

AD-associated gene expression changes might occur in only a small subpopulation of microglia that are in closer proximity to Aβ plaques. In bulk RNAseq, gene expression changes in such a microglial subpopulation might remain undetected as bulk RNAseq provides the average gene expression profile of all microglia in a sample. To address this possibility, we employed scRNAseq to assess AD-associated gene expression changes in individual microglia. Microglia derived from the LPS region were analyzed with a median of 714 cells per donor from four CTR, five CTR+, one donor with MCI, and four AD donors. Clustering analysis identified seven microglia subsets, indicating heterogeneity in microglia transcriptomes (Figure 3A). The sequencing depth and number of uniquely detected genes were variable between donors (Supplementary Figures S4A,B), but comparable across clusters (Supplementary Figures S4D,E). The percentage of mitochondrial transcripts detected were comparable across donors and clusters except for the smallest cluster 6 (*n* = 29 cells; Supplementary Figures S4C,F). Each donor contributed cells to most clusters, including the larger clusters 0–2 (Figure 3B). The absence of the formation of donor-specific clusters indicates that cluster formation was likely not influenced by donor-specific effects. The expression of cell type–specific markers across all clusters showed that all analyzed cells were microglia (*ITGAX*) without contamination of neurons (*RBFOX3*), oligodendrocytes (*MOG*), astrocytes (*GFAP*), circulating monocytes (*CCR2*), and erythrocytes (*HBA1*; Figure 3C).

Differential gene expression analysis between any cluster compared to all other clusters was used to identify cluster-enriched genes (Supplementary Table S6). The small clusters 3–6 contained relatively few cells of the total microglia population (0.3%–1.6%) and were marked by a very low number of unique cluster-enriched genes (Supplementary Table S6). Notably, cluster-enriched genes in the small clusters were expressed in more than 75% of the cells in the small clusters, whereas cluster-enriched genes were detected in ~30% of the cells in the large clusters 0–2. In addition, expression levels of cluster-enriched genes were higher in the smaller clusters than in the larger clusters (Figure 3D, Supplementary Table S7). This very frequent expression of few genes with strong enrichment could have resulted in the formation of the smaller clusters 3–6. The small clusters showed a unique enrichment for *SMIM11A* and -*B* (cluster 3); *MEF2C*, *GPR89A*, and -*B* (cluster 4); *SERF1A* and -*B* (cluster 5); and *FRG1* and *FRG1CP* (cluster 6; Figure 3D, Supplementary Tables S6, S7). Gene ontology (GO) enrichment analysis revealed no biological annotation associated with the small clusters.

Microglia in cluster 0 were uniquely enriched in the genes *AXL, CLEC7A, CYBB* (Figure 3D, Supplementary Tables S6, S7). These genes were associated with a hyper-responsive inflammatory phenotype conserved in aging and neurodegenerative-related mouse models that is involved in functions, such as phagocytosis (Holtman et al., 2015). In addition, microglia in cluster 0 were uniquely enriched in cytoskeleton-related genes (*FGD2, ACTB, SRGAP2*; Figure 3D, Supplementary Tables S6, S7). These genes were associated with GO terms involved in locomotion, endocytosis, and filopodium assembly (Supplementary Figure S5, Supplementary Table S8). Microglia in cluster 1 uniquely expressed genes involved in transcriptional activity (*ZNF302, NEAT1*, and *ANKRD11*; Figure 3D, Supplementary Tables S6, S7), and the associated GO terms included RNA splicing (Supplementary Figure S5, Supplementary Table S8). Microglia in cluster 2 were uniquely enriched in genes associated with neurodegenerative diseases (*TREM2*, *GLUL*, *S100A*; Keren-Shaul et al., 2017; Krasemann et al., 2017; Cristóvaõ and Gomes, 2019) and immune activated microglia (*CD63*, *HLA* genes, *CD14*, *TSPO*; Beschorner et al., 2002; Kamphuis et al., 2016; Beckers et al., 2018) and in ribosomal genes (*RPL* genes; Figure 3D, Supplementary Tables S6, S7). Associated GO terms included protein targeting and active immune response (Supplementary Figure S5, Supplementary Table S8). Cells of all clusters showed higher expression of DAM genes compared to homeostatic microglia markers (Figure 3D, Supplementary Table S7).

To determine if expression of the DAM-associated gene set was altered in our human microglia clusters, average expression of the DAM gene set was calculated per cell. Mean expression per cluster was compared to all other clusters. The mean expression level of the DAM gene set was significantly reduced in clusters 0 and 1 and significantly increased in cluster 2 (Figure 3E, Supplementary Table S9). To validate these observations, the same analysis was performed for two other gene sets. The first was obtained from gene expression modules in myeloid cells from a comparison between AD tissue and multiple neurodegeneration-related mouse models (Friedman et al., 2018) and the second from a study investigating single-nucleus transcriptomes in the prefrontal cortex of human AD donors (Mathys et al., 2019). Similar to the results of the DAM gene set, cells in cluster 1 showed a significant decrease in mean gene expression of the myeloid neurodegeneration-related and human AD-related single-nucleus gene sets (Figure 3E, Supplementary Table S9). Cluster 2 showed a significant increase in mean gene expression of the DAM gene set and the human AD-related single-nucleus gene set (Figure 3E, Supplementary Table S9). Although statistically significant, mean expression level changes are likely too small to be biologically relevant. Together with the identified cluster-enriched genes and associated GO terms, cluster 2 might have increased immune activated gene expression. However, cluster 2 was not enriched in AD-donor derived microglia (Figure 3B), indicating that the immune activated phenotype of this microglial subpopulation is not unique to AD pathology.

Taken together, scRNAseq analysis of approximately 10,000 microglia from 14 donors identified subtle microglial heterogeneity but no AD-specific microglia subpopulation.

### Microglia Diversity but No DAM-Like Cluster in an Individual MCI and AD Donor With High Microglial Cell Numbers

The proportion of microglia associated with AD pathology in the human brain might be relatively low and could potentially be missed by the more limited cell numbers analyzed by bc-Smart-seq2 expression profiling. Therefore, ~3,000 microglia per donor from two donors were analyzed using scRNAseq with the 10× Genomics platform, leading to a considerably higher cell number compared to the 714 microglia per donor analyzed with bc-Smart-seq2. We hypothesized that different microglial subpopulations, such as plaque-associated and homeostatic microglia populations, are present in the AD brain and that analysis of a higher number of microglia from the same donor would allow for the identification of relatively small subpopulations. To prevent donor-associated variables (sex, age, postmortem delay, tissue quality, etc.) affecting microglia clustering, each donor was analyzed individually: 2,881 cells were analyzed from donor 1: AD, female, 81 years, LPS tissue with high Aβ burden, and modest but visible PHF-tau protein (donor #2018-135, Supplementary Table S2), and 3,079 single cells were analyzed from donor 2: MCI donor, female, 77 years old, LPS tissue with moderate Aβ pathology, and high levels of PHF-tau deposits (donor #2019-010, Supplementary Table S2).

**Figure 4:**
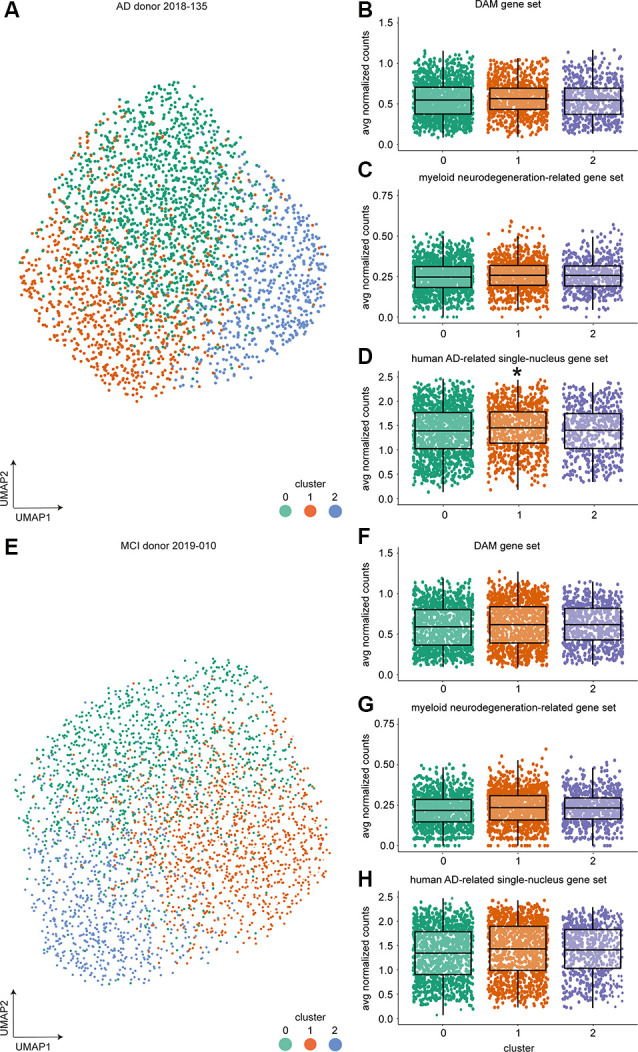
Single-cell expression profiling of microglia from individual donors identified three clusters but no DAM-like cluster using 10× Genomics. **(A)** UMAP visualization and unsupervised clustering of microglia from AD donor 2018-135. **(B–D)** Average expression of the mouse DAM **(B)**, myeloid neurodegeneration-related **(C)**, and human AD-related single-nucleus **(D)** gene set expression per cell across clusters for AD donor 2018-135 (multiple linear regression with Bonferroni correction, *indicate *p*-value < 0.001). **(E)** UMAP visualization and unsupervised clustering of MCI donor 2019-010. **(F–H)** Average expression of the mouse DAM **(F)**, myeloid neurodegeneration-related **(G)** and human AD-related single-nucleus **(H)** gene set expression per cell across clusters for MCI donor 2019-010. Horizontal lines in the boxplots represent the mean; the lower and upper hinges represent the 25th and 75th percentiles. Abbreviations: AD, Alzheimer’s Disease; avg, average; CPM, counts per million; CTR, Control; CTR+, Control with Aβ plaques and/or hyperphosphorylated tau; DAM, disease-associated microglia; MCI, mild cognitive impairment; UMAP, uniform manifold approximation and projection.

In both donors, three microglial clusters were identified (Figures 4A,E, Supplementary Tables S10, S11). To identify whether cells in any of these clusters were enriched in the expression of genes associated with neurodegenerative diseases, we averaged gene expression of the mouse DAM (Keren-Shaul et al., 2017), the myeloid neurodegeneration-related (Friedman et al., 2018), and the human AD-related single-nucleus (Mathys et al., 2019) gene sets per cell. Next, mean expression per cluster was compared to all other clusters (Figures 4B–D,F–H, Supplementary Table S9). A significant increase in mean gene expression of the human AD-associated single-nucleus gene set was observed in cluster 1 microglia of the AD (Figure 4D) but not the MCI donor (Figure 4H). To summarize, although a high number of microglia was analyzed per donor, none of the three clusters per donor could consistently be related to known microglia gene expression changes associated with AD.

## Discussion

In this study, we aimed to identify transcriptomic changes in human microglia at the end stage of AD by applying both bulk and scRNAseq of microglia isolated from acute postmortem CNS tissue. In parietal and frontal cortex, we analyzed microglia as bulk samples allowing the most sensitive detection of small gene expression changes. Here, transcriptomic differences between AD and CTR were not detected but were present between AD and CTR+. Possibly, the difference between CTR vs. AD could not be detected in our data set due to limited sample numbers and/or relatively small changes. Alternatively, CTR+ donors with amyloid-beta plaques and tau pathology have different transcriptomic changes in microglia than CTR donors with respect to AD. Disease-associated genes identified previously in AD mouse models were not enriched in bulk human AD microglia. Next, single-cell sequencing analysis was applied to detect microglial subtypes that possibly consist of low cell numbers and might be undetected in the average transcriptome obtained by bulk RNAseq. In single microglia transcriptomes, the relative contribution to microglia clusters did not differ between AD and control donors, when using the bc-Smart-seq2 protocol. In addition, expression of genes related to neurodegenerative disease from previous studies (Keren-Shaul et al., 2017; Friedman et al., 2018; Mathys et al., 2019) were not altered with meaningful effect sizes in any cluster.

The neurodegenerative disease-associated microglial subtype originally described by Krasemann et al. (2017) and similarly described by Keren-Shaul et al. (2017) represented a relatively small fraction of the total microglia population. To rule out that the lack of a cluster associated with AD pathology in our bc-Smart-seq2 data was due to the analysis of low cell numbers, a higher number of microglia from two donors were single-cell sequenced using the 10× Genomics platform. Clustering was performed per donor to avoid donor variation that might mask such a potential cluster. None of the clusters identified with 10× Genomics scRNAseq were consistently enriched in mean expression of gene sets related to neurodegenerative diseases. In conclusion, a DAM-like microglial subtype was absent in AD donors profiling relatively high cell numbers.

When comparing the clustering results of the 10× Genomics and bc-Smart-seq2 scRNAseq techniques, differences were observed. Clusters 0–2 in the bc-Smart-seq2 data contain the vast majority of microglia and are most similar to the three clusters identified per donor in the 10× Genomics data set. The smaller clusters in the bc-Smart-seq2 data (4–8) were not identified in the microglia profiled by 10× Genomics and are possibly associated with the plate-based protocol as we observed similar small clusters in a different bc-Smart-seq2 data set from our group (unpublished results).

There are multiple possible explanations for the absence of AD-associated changes in bulk and single-cell microglia acutely isolated from postmortem human brain tissue. Limitations of this study are the relatively small sample sizes, especially in the CTR group. In addition, interindividual differences between donors might mask gene expression differences in the bc-Smart-seq2 study. The presence of comorbidity, medication, and varying postmortem delay (time from death to autopsies) might interfere with AD-specific effects on the microglial transcriptome. However, these factors are difficult to avoid in human postmortem data. Another explanation for the lack of AD-related transcripts in bulk and single-cell microglia could be that the relevant microglia associated with AD plaques are more vulnerable to the isolation procedure. This would imply that, using conventional isolation and sorting of microglia would enrich a population of cells that are not related to AD pathology. Streit and colleagues first introduced the concept of dystrophic microglia that occur around neuronal structures positive for hyperphosphorylated tau protein (Streit et al., 2004, 2009) and were later found to also occur around Aβ plaques (Streit et al., 2018). Possibly, dystrophic microglia and microglia embedded inside the Aβ plaque are more vulnerable and, therefore, preferentially lost during FACS gating of live, single cells from human brain tissue.

When comparing AD mouse models to human AD, the distinction between parenchymal and plaque-associated microglia might be more pronounced for amyloid mouse models than for human end-stage AD samples. In transgenic amyloid mouse models, especially 5xFAD mice, Aβ is overexpressed in a nonphysiological manner. This results in very fast Aβ plaque formation and, at end stages, a much higher plaque burden in AD mouse models than in the human AD brain (Drummond and Wisniewski, 2017; Liu et al., 2017). Transgenic mouse models lack regional brain atrophy and show less widespread neurodegeneration than human AD cases (Drummond and Wisniewski, 2017). Possibly, compared to plaque-associated microglia, parenchymal microglia are less affected in amyloid mouse models than in human AD brain tissue. Furthermore, single human microglia studies will most likely require much larger cell numbers to capture sufficient plaque-associated human microglia than studies using AD mouse models. Additionally, interindividual variation will influence human microglia transcriptomes more than mouse microglia transcriptomes. Together, these factors might lead to a more pronounced change between parenchymal and plaque-associated microglia in amyloid mouse models than in human AD samples.

DAMs were not only associated with neurodegenerative diseases, but also with natural aging (Keren-Shaul et al., 2017; Krasemann et al., 2017). This was confirmed in a study identifying an AppNL-G-F-associated microglia subpopulation, activated response microglia, which overlap with DAMs (Sala Frigerio et al., 2019). Activated response microglia already comprised a few percent of microglia in the brains of wild-type mice at a young age and evolve naturally with aging (Sala Frigerio et al., 2019). Furthermore, a consensus gene expression network module co-occurring both during aging and neurodegeneration was previously described (Holtman et al., 2015). The described module included DAM signature genes, such as *Csf1*, *Spp1*, *Apoe*, *Axl*, *B2m*, *Ctsz*, *Cd9*, *Cstb*, and *Cst7*. Biological annotation of module-specific genes included phagosome and lysosomal pathways (Holtman et al., 2015), functions associated with DAMs (Keren-Shaul et al., 2017). Taken together, this suggests the presence of DAM-like microglia could be expected, albeit at low levels, in aged controls as well as AD donor-derived microglia.

It is still an unresolved question whether a subpopulation resembling DAMs exist among human microglia. Three other studies previously addressed this question. Olah et al. (2018) observed 23 clusters of human microglia, where five out of 23 clusters were enriched for DAM signature genes. Three of the 15 donors suffered from AD pathology, making it difficult to connect their microglia subpopulations with AD-induced gene expression changes. Srinivasan et al. (2019) investigated frozen myeloid cells from AD brain tissue and observed that, from the 100 DAM genes, only expression of *APOE* did change in myeloid cells from AD donors compared to controls. Mathys et al. (2019) used single-nuclei sequencing and subclustered ~2,400 microglia of 48 donors into four subpopulations. They highlighted the *microglia1* cluster as AD-pathology-associated human microglia. From the 257 DAM genes investigated by Mathys et al. (2019), 28 were overlapping with marker genes for the *microglia1* cluster, and 16 of these 28 overlapping marker genes were ribosomal genes. Although this reveals a starting point, a larger-scale investigation of microglia nuclei is needed to identify AD-associated microglia subpopulations in humans.

Single nucleus gene expression faithfully recapitulates cellular gene expression profiles (Lake et al., 2017; Gerrits et al., 2020). Therefore, single-nucleus sequencing offers an alternative to scRNAseq that is especially useful in tissues from which recovering intact cells is difficult (Grindberg et al., 2013; Lake et al., 2017; Gerrits et al., 2020). An important advantage of single-nucleus sequencing is the possibility to use frozen samples from brain banks containing large, well-characterized donor cohorts. For example, a donor cohort to differentiate the effects of natural aging and AD pathology would be possible by comparing aged-matched (young) control donors to early-onset familial AD cases using frozen samples from brain banks. In the future, single-nucleus sequencing of microglia, including tissues of early, presymptomatic stages of AD will be most promising to potentially identify microglia biomarkers for AD.

## Data Availability Statement

The data sets generated for this study can be found in the Gene Expression Omnibus, GSE146639.

## Ethics Statement

The studies involving human participants were reviewed and approved by Ethics committee of the VU University Medical Center (VUMC, Amsterdam, The Netherlands). The patients/participants provided their written informed consent to participate in this study.

## Author Contributions

EB, BE, EH, JM, and SK were responsible for the overall conception of the project and provided supervision. QJ, AA, and LK conducted the experimental work and/or analyzed the data, prepared the figures, and wrote the manuscript. EG and RD assisted with sample processing, data discussion, and performed the immunohistochemistry. NB, AM, MD, MW, AW, SX, KB, TM, JM, and RD assisted with maintenance of availability of reagents, sample processing, data discussion, optimization issues, and/or data analysis. All authors contributed to the article and approved the submitted version.

## Conflict of Interest

MW, AW, SX, TM and KB were full time employees of Abbvie during the time of the studies. The remaining authors declare that the research was conducted in the absence of any commercial or financial relationships that could be construed as a potential conflict of interest.

## Footnotes

^1^https://www.brainbank.nl/

## Supplementary Material

FIGURE S1 Donor classification based on immunohistochemistry. Representative immunohistochemical images of Aß, PHF-tau, and IBA1 staining of consecutive sections from the LPS of the three donor groups (CTR, CTR+, AD). Scale bar = 300 μm, scale bar inset = 50 μm. Abbreviations: AD, clinically diagnosed Alzheimer’s Disease with Aβ plaques and/or hyperphosphorylated tau; CTR, Control; CTR+, Control with Aβ plaques and/or hyperphosphorylated tau; LPS, superior parietal lobe.

FIGURE S2 Quality metrics of bulk RNAseq data. **(A–D)** Total reads **(A)**, uniquely mapped reads **(B)**, exonic reads **(C)**, RNA Integrity (RIN) values **(D)** are depicted for all microglia samples (CTR, CTR+, AD) of both brain regions (GFS, LPS). Abbreviations: AD, clinically diagnosed Alzheimer’s Disease with Aβ plaques and/or hyperphosphorylated tau; CTR, Control; CTR+, Control with Aβ plaques and/or hyperphosphorylated tau; GFS, superior frontal gyrus; LPS, superior parietal lobe; M, million; RIN, RNA integrity value.

FIGURE S3 Differentially expressed genes between parietal (LPS) and frontal (GFS) brain regions. Selected examples of expression levels of genes differentially expressed between LPS and GFS shown in log_2_ CPM (likelihood ratio test; *JAML* ***FDR < 0.001, *CST7* ***FDR < 0.001 and *TREM1* ***FDR < 0.001, *AC011451.1 ** FDR = 0.04). Abbreviations: CPM, counts per million; GFS, superior frontal gyrus; LPS, superior parietal lobe.

FIGURE S4 Bc-Smart-seq2 single-cell RNA sequencing quality of donors and clusters. **(A–F)** Boxplots displaying the number of detected UMIs, unique genes and percentage of mitochondrial transcripts in single cells per donor **(A,B,D)** and per cluster **(D–F)**. Abbreviations: AD, clinically diagnosed Alzheimer’s Disease with Aβ plaques and/or hyperphosphorylated tau; CTR, Control; CTR+, Control with Aβ plaques and/or hyperphosphorylated tau, UMI; unique molecular identifier.

FIGURE S5 Biological annotation of marker genes for each cluster. Dot plots displaying the top 10 gene ontology terms per cluster identified in bc-Smart-seq2 single-cell data. Gene ontology terms were ordered on gene count. Color indicates the adjusted *p*-value. No GO terms were significantly associated with genes of clusters 5 and 6.

TABLE S1 Detailed donor information of bulk sequenced samples.

TABLE S2 Detailed donor information of single-cell sequenced samples.

TABLE S3 Differential gene expression analysis of male vs. female bulk microglia samples.

TABLE S4 Differential gene expression analysis of LPS vs. GFS bulk microglia samples (related to Figure S3).

TABLE S5 Differential gene expression analysis of CTR+/CTR vs. AD bulk microglia samples (related to Figure 2B).

TABLE S6 Cluster-enriched genes of the identified seven clusters with bc-Smart-seq2 single-cell RNA sequencing.

TABLE S7 Average scaled expression for and percentage of cells expressing selected genes in clusters identified with bc-Smart-seq2 single-cell RNA sequencing (related to Figure 3D).

TABLE S8 GO terms associated with the cluster-enriched genes of bc-Smart-seq2 single-cell RNA sequencing (related to Supplementary Figure S5).

TABLE S9 Statistics of the average gene set expression changes in single-cell clusters (related to Figures 3E, 4B–D,F–H).

TABLE S10 Cluster-enriched genes of the identified three clusters of donor 2018-135 with 10× Genomics single-cell RNA sequencing.

TABLE S11 Cluster-enriched genes of the identified three clusters of donor 2019-10 with 10× Genomics single-cell RNA sequencing.

## References

[B1] Bakshi P. Margenthaler E. Reed J. Crawford F. Mullan M. (2011). Depletion of CXCR2 inhibits γ-secretase activity and amyloid-β production in a murine model of Alzheimer’s disease. Cytokine 53, 163–169. 10.1016/j.cyto.2010.10.008 21084199

[B2] Beckers L. Ory D. Geric I. Declercq L. Koole M. Kassiou M. (2018). Increased expression of translocator protein (TSPO) marks pro-inflammatory microglia but does not predict neurodegeneration. Mol. Imaging Biol. 20, 94–102. 10.1007/s11307-017-1099-1 28695372

[B3] Beschorner R. Nguyen T. D. Gözalan F. Pedal I. Mattern R. Schluesener H. J.. (2002). CD14 expression by activated parenchymal microglia/macrophages and infiltrating monocytes following human traumatic brain injury. Acta Neuropathol. 103, 541–549. 10.1007/s00401-001-0503-7 12012085

[B4] Bogie J. F. J. Boelen E. Louagie E. Delputte P. Elewaut D. van Horssen J.. (2018). CD169 is a marker for highly pathogenic phagocytes in multiple sclerosis. Mult. Scler. J. 24, 290–300. 10.1177/1352458517698759 28277099

[B5] Bradburn S. McPhee J. Bagley L. Carroll M. Slevin M. Al-Shanti N.. (2018). Dysregulation of C-X-C motif ligand 10 during aging and association with cognitive performance. Neurobiol. Aging 63, 54–64. 10.1016/j.neurobiolaging.2017.11.009 29223680 PMC5805841

[B6] Butler A. Hoffman P. Smibert P. Papalexi E. Satija R. (2018). Integrating single-cell transcriptomic data across different conditions, technologies and species. Nat. Biotechnol. 36, 411–420. 10.1038/nbt.4096 29608179 PMC6700744

[B7] Cristóvaõ J. S. Gomes C. M. (2019). S100 proteins in Alzheimer’s disease. Front. Neurosci. 13:463. 10.3389/fnins.2019.00463 31156365 PMC6532343

[B8] Drummond E. Wisniewski T. (2017). Alzheimer’s disease: experimental models and reality. Acta Neuropathol. 133, 155–175. 10.1007/s00401-016-1662-x 28025715 PMC5253109

[B9] Friedman B. A. Srinivasan K. Ayalon G. Meilandt W. J. Lin H. Huntley M. A.. (2018). Diverse brain myeloid expression profiles reveal distinct microglial activation states and aspects of Alzheimer’s disease not evident in mouse models. Cell Rep. 22, 832–847. 10.1016/j.celrep.2017.12.066 29346778

[B10] Galatro T. F. Holtman I. R. Lerario A. M. Vainchtein I. D. Brouwer N. Sola P. R.. (2017a). Transcriptomic analysis of purified human cortical microglia reveals age-associated changes. Nat. Neurosci. 20, 1162–1171. 10.1038/nn.4597 28671693

[B11] Galatro T. F. Vainchtein I. D. Brouwer N. Boddeke E. W. G. M. Eggen B. J. L. (2017b). Isolation of microglia and immune infiltrates from mouse and primate central nervous system. Methods Mol. Biol. 1559, 333–342. 10.1007/978-1-4939-6786-5_23 28063055

[B12] Galimberti D. Schoonenboom N. Scheltens P. Fenoglio C. Bouwman F. Venturelli E.. (2006). Intrathecal chemokine synthesis in mild cognitive impairment and Alzheimer disease. Arch Neurol. 63, 538–543. 10.1001/archneur.63.4.538 16606766

[B13] George N. I. Chang C. W. (2014). DAFS: a data-adaptive flag method for RNA-sequencing data to differentiate genes with low and high expression. BMC Bioinformatics 15:92. 10.1186/1471-2105-15-92 24685233 PMC4098771

[B14] Gerrits E. Heng Y. Boddeke H. W. G. M. Eggen B. J. L. (2020). Transcriptional profiling of microglia; current state of the art and future perspectives. Glia 68, 740–755. 10.1002/glia.23767 31846124 PMC7064956

[B15] Griffiths J. A. Richard A. C. Bach K. Lun A. T. L. Marioni J. C. (2018). Detection and removal of barcode swapping in single-cell RNA-seq data. Nat. Commun. 9:2667. 10.1038/s41467-018-05083-x 29991676 PMC6039488

[B16] Grindberg R. V. Yee-Greenbaum J. L. McConnell M. J. Novotny M. O’Shaughnessy A. L. Lambert G. M.. (2013). RNA-sequencing from single nuclei. Proc. Natl. Acad. Sci. U S A 110, 19802–19807. 10.1073/pnas.1319700110 24248345 PMC3856806

[B17] Holtman I. R. Raj D. D. Miller J. A. Schaafsma W. Yin Z. Brouwer N.. (2015). Induction of a common microglia gene expression signature by aging and neurodegenerative conditions: a co-expression meta-analysis. Acta Neuropathol. Commun. 3:31. 10.1186/s40478-015-0203-5 26001565 PMC4489356

[B18] Hyman B. T. Trojanowski J. Q. (1997). Editorial on consensus recommendations for the postmortem diagnosis of Alzheimer disease from the National Institute on Aging and the Reagan Institute Working Group on diagnostic criteria for the neuropathological assessment of Alzheimer disease. J. Neuropathol. Exp. Neurol. 56, 1095–1097. 10.1097/00005072-199710000-00002 9329452

[B19] Kamphuis W. Kooijman L. Schetters S. Orre M. Hol E. M. (2016). Transcriptional profiling of CD11c-positive microglia accumulating around amyloid plaques in a mouse model for Alzheimer’s disease. Biochim. Biophys. Acta 1862, 1847–1860. 10.1016/j.bbadis.2016.07.007 27425031

[B20] Keren-Shaul H. Spinrad A. Weiner A. Matcovitch-Natan O. Dvir-Szternfeld R. Ulland T. K.. (2017). A unique microglia type associated with restricting development of Alzheimer’s disease. Cell 169, 1276.e17–1290.e17. 10.1016/j.cell.2017.05.018 28602351

[B21] Kim D. Langmead B. Salzberg S. L. (2015). HISAT: a fast spliced aligner with low memory requirements. Nat. Methods 12, 357–360. 10.1038/nmeth.3317 25751142 PMC4655817

[B22] Krasemann S. Madore C. Cialic R. Baufeld C. Calcagno N. El Fatimy R.. (2017). The TREM2-APOE pathway drives the transcriptional phenotype of dysfunctional microglia in neurodegenerative diseases. Immunity 47, 566.e9–581.e9. 10.1016/j.immuni.2017.08.008 28930663 PMC5719893

[B23] Lake B. B. Codeluppi S. Yung Y. C. Gao D. Chun J. Kharchenko P. V.. (2017). A comparative strategy for single-nucleus and single-cell transcriptomes confirms accuracy in predicted cell-type expression from nuclear RNA. Sci. Rep. 7:6031. 10.1038/s41598-017-04426-w 28729663 PMC5519641

[B24] Lake B. Shen R. Ronaghi M. Fan J. Wang W. Zhang K. (2016). Neuronal subtypes and diverstiy revealed by single-nucleus RNA sequencing of human brain. Science 352, 1586–1590. 10.1126/science.aaf1204 27339989 PMC5038589

[B25] Liao Y. Smyth G. K. Shi W. (2014). FeatureCounts: an efficient general purpose program for assigning sequence reads to genomic features. Bioinformatics 30, 923–930. 10.1093/bioinformatics/btt656 24227677

[B26] Liu P. Reichl J. H. Rao E. R. McNellis B. M. Huang E. S. Hemmy L. S.. (2017). Quantitative comparison of dense-core amyloid plaque accumulation in amyloid-β protein precursor transgenic mice. J. Alzheimers Dis. 56, 743–761. 10.3233/JAD-161027 28059792 PMC5272806

[B27] Mathys H. Davila-Velderrain J. Peng Z. Gao F. Mohammadi S. Young J. Z.. (2019). Single-cell transcriptomic analysis of Alzheimer’s disease. Nature 570, 332–337. 10.1101/164889 31042697 PMC6865822

[B28] Mhatre S. D. Tsai C. A. Rubin A. J. James M. L. Andreasson K. I. (2015). Microglial malfunction: the third rail in the development of Alzheimer’s disease. Trends Neurosci. 38, 621–636. 10.1016/j.tins.2015.08.006 26442696 PMC4670239

[B29] Nott A. Holtman I. R. Coufal N. G. Schlachetzki J. C. M. Yu M. Hu R.. (2019). Brain cell type-specific enhancer-promoter interactome maps and disease-risk association. Science 366, 1134–1139. 10.1126/science.aay0793 31727856 PMC7028213

[B30] Olah M. Menon V. Habib N. Taga M. Yung C. Elyaman W. (2018). A single cell-based atlas of human microglial states reveals associations with neurological disorders and histopathological features of the aging brain. bioRxiv [Preprint]. 343780 10.1101/343780

[B31] Picelli S. Faridani O. R. Bjorklund A. K. Winberg G. Sagasser S. Sandberg R. (2014). Full-length RNA-seq from single cells using Smart-seq2. Nat. Protoc. 9, 171–181. 10.1038/nprot.2014.006 24385147

[B32] Robinson M. D. Mccarthy D. J. Smyth G. K. (2010). edgeR: a bioconductor package for differential expression analysis of digital gene expression data. Bioinformatics 26, 139–140. 10.1093/bioinformatics/btp616 19910308 PMC2796818

[B33] Rogers J. Lue L.-F. (2001). Microglial chemotaxis, activation and phagocytosis of amyloid β-peptide as linked phenomena in Alzheimer’s disease. Neurochem. Int. 39, 333–340. 10.1016/s0197-0186(01)00040-7 11578768

[B34] Ryu J. K. Cho T. Choi H. B. Jantaratnotai N. McLarnon J. G. (2015). Pharmacological antagonism of interleukin-8 receptor CXCR2 inhibits inflammatory reactivity and is neuroprotective in an animal model of Alzheimer’s disease. J. Neuroinflammation 12:144. 10.1186/s12974-015-0339-z 26255110 PMC4529987

[B35] Sala Frigerio C. Wolfs L. Fattorelli N. Thrupp N. Voytyuk I. Schmidt I.. (2019). The major risk factors for Alzheimer’s disease: age, sex and genes modulate the microglia response to Aβ plaques. Cell Rep. 27, 1293.e6–1306.e6. 10.1016/j.celrep.2019.03.099 31018141 PMC7340153

[B36] Sarlus H. Heneka M. T. (2017). Microglia in Alzheimer’s disease. J. Clin. Invest. 127, 3240–3249. 10.1172/JCI90606 28862638 PMC5669553

[B37] Smith T. Heger A. Sudbery I. (2017). UMI-tools: modelling sequencing error in unique molecular identifiers to improve quantification. Genome Res. 27, 491–499. 10.1101/gr.209601.116 28100584 PMC5340976

[B38] Spangenberg E. Severson P. L. Hohsfield L. A. Crapser J. Zhang J. Burton E. A.. (2019). Sustained microglial depletion with CSF1R inhibitor impairs parenchymal plaque development in an Alzheimer’s disease model. Nat. Commun. 10:3758. 10.1038/s41467-019-11674-z 31434879 PMC6704256

[B39] Srinivasan K. Friedman B. A. Etxeberria A. Huntley M. A. van der Brug M. P. Foreman O. (2019). Alzheimer’s patient brain myeloid cells exhibit enhanced aging and unique transcriptional activation. bioRxiv [Preprint]. 610345 10.1101/610345 PMC7422733 32610143

[B40] Streit W. J. Braak H. Del Tredici K. Leyh J. Lier J. Khoshbouei H.. (2018). Microglial activation occurs late during preclinical Alzheimer’s disease. Glia 66, 2550–2562. 10.1002/glia.23510 30417428

[B41] Streit W. J. Braak H. Xue Q.-S. Bechmann I. (2009). Dystrophic (senescent) rather than activated microglial cells are associated with tau pathology and likely precede neurodegeneration in Alzheimer’s disease. Acta Neuropathol. 118, 475–485. 10.1007/s00401-009-0556-6 19513731 PMC2737117

[B42] Streit W. J. Sammons N. W. Kuhns A. J. Sparks D. L. (2004). Dystrophic microglia in the aging human brain. Glia 45, 208–212. 10.1002/glia.10319 14730714

[B43] The ENCODE Consortium (2016). ENCODE guidelines and best practices for RNA-Seq: revised December 2016. Available online at: https://www.encodeproject.org/documents/cede0cbe-d324-4ce7-ace4-f0c3eddf5972/@@download/attachment/ENCODE%20Best%20Practices%20for%20RNA_v2.pdf. Accessed June 16, 2020.

[B44] Ulrich J. D. Finn M. B. Wang Y. Shen A. Mahan T. E. Jiang H.. (2014). Altered microglial response to Aβ plaques in APPPS1–21 mice heterozygous for TREM2. Mol. Neurodegener. 9:20. 10.1186/1750-1326-9-20 24893973 PMC4049806

[B45] Uniken Venema W. T. Voskuil M. D. Vila A. V. van der Vries G. Jansen B. H. Jabri B.. (2019). Single-cell RNA sequencing of blood and ileal T cells from patients with Crohn’s disease reveals tissue-specific characteristics and drug targets. Gastroenterology 156, 812.e22–815.e22. 10.1053/j.gastro.2018.10.046 30391472 PMC6759855

[B46] Veenstra M. Ransohoff R. M. (2012). Chemokine receptor CXCR2: physiology regulator and neuroinflammation controller? J. Neuroimmunol. 246, 1–9. 10.1016/j.jneuroim.2012.02.016 22445294 PMC3335977

[B47] Venegas C. Kumar S. Franklin B. S. Dierkes T. Brinkschulte R. Tejera D.. (2017). Microglia-derived ASC specks cross-seed amyloid-β in Alzheimer’s disease. Nature 552, 355–361. 10.1038/nature25158 29293211

[B48] Xia M. Q. Hyman B. T. (2002). GROα/KC, a chemokine receptor CXCR2 ligand, can be a potent trigger for neuronal ERK1/2 and PI-3 kinase pathways and for tau hyperphosphorylation-a role in Alzheimer’s disease? J. Neuroimmunol. 122, 55–64. 10.1016/s0165-5728(01)00463-5 11777543

[B49] Yin Z. Raj D. Saiepour N. Van Dam D. Brouwer N. Holtman I. R.. (2017). Immune hyperreactivity of Aβ plaque-associated microglia in Alzheimer’s disease. Neurobiol. Aging 55, 115–122. 10.1016/j.neurobiolaging.2017.03.021 28434692

[B50] Yu G. Wang L.-G. Han Y. He Q.-Y. (2012). clusterProfiler: an r package for comparing biological themes among gene clusters. OMICS 16, 284–287. 10.1089/omi.2011.0118 22455463 PMC3339379

[B51] Zhang B. Gaiteri C. Bodea L.-G. Wang Z. McElwee J. Podtelezhnikov A. A.. (2013). Integrated systems approach identifies genetic nodes and networks in late-onset Alzheimer’s disease. Cell 153, 707–720. 10.1016/j.cell.2013.03.030 23622250 PMC3677161

[B52] Zhong S. Zhang S. Fan X. Wu Q. Yan L. Dong J.. (2018). A single-cell RNA-seq survey of the developmental landscape of the human prefrontal cortex. Nature 555, 524–528. 10.1038/nature25980 29539641

