# Supplementary figures and images for: Profiling Microglia From Alzheimer’s Disease Donors and Non-demented Elderly in Acute Human Postmortem Cortical Tissue
Source: Front Mol Neurosci. 2020 Oct 28;13:134. doi: 10.3389/fnmol.2020.00134 (PMC7655794; doi:10.3389/fnmol.2020.00134)

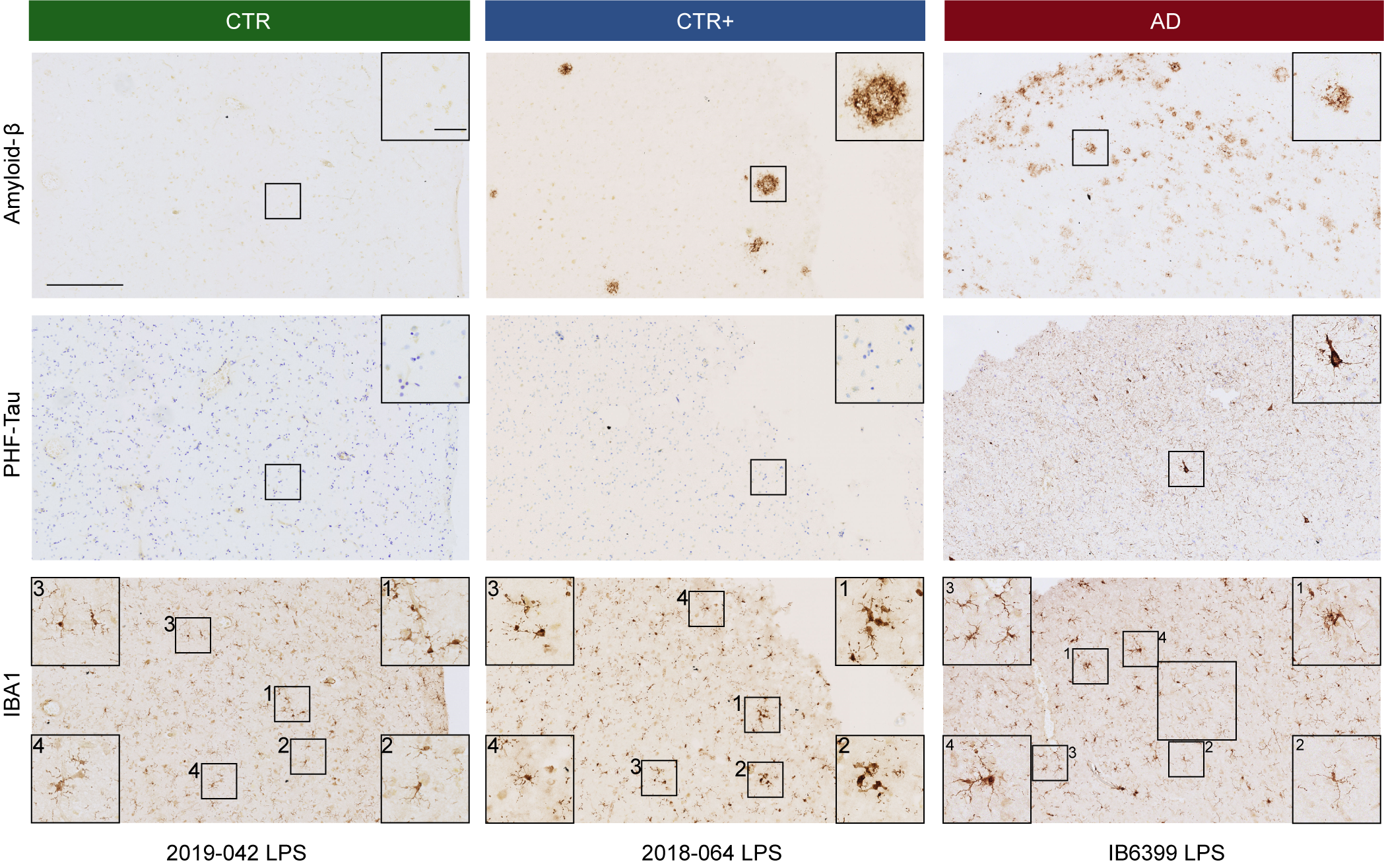

Supplement: FIGURE S1 — Donor classification based on immunohistochemistry. Representative immunohistochemical images of Aß, PHF-tau, and IBA1 staining of consecutive sections from the LPS of the three donor groups (CTR, CTR+, AD). Scale bar = 300 μm, scale bar inset = 50 μm. Abbreviations: AD, clinically diagnosed Alzheimer’s Disease with Aβ plaques and/or hyperphosphorylated tau; CTR, Control; CTR+, Control with Aβ plaques and/or hyperphosphorylated tau; LPS, superior parietal lobe. [file Image_1.TIF]

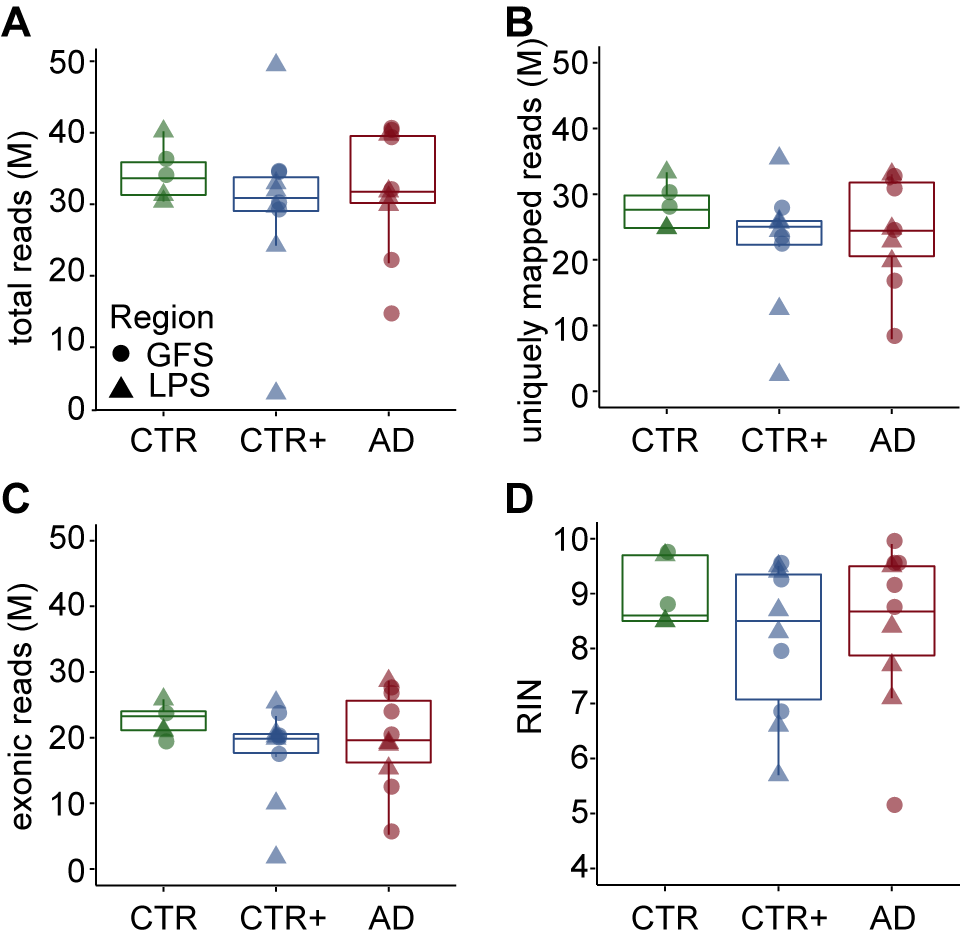

Supplement: FIGURE S2 — Quality metrics of bulk RNAseq data. (A–D) Total reads (A), uniquely mapped reads (B), exonic reads (C), RNA Integrity (RIN) values (D) are depicted for all microglia samples (CTR, CTR+, AD) of both brain regions (GFS, LPS). Abbreviations: AD, clinically diagnosed Alzheimer’s Disease with Aβ plaques and/or hyperphosphorylated tau; CTR, Control; CTR+, Control with Aβ plaques and/or hyperphosphorylated tau; GFS, superior frontal gyrus; LPS, superior parietal lobe; M, million; RIN, RNA integrity value. [file Image_2.TIF]

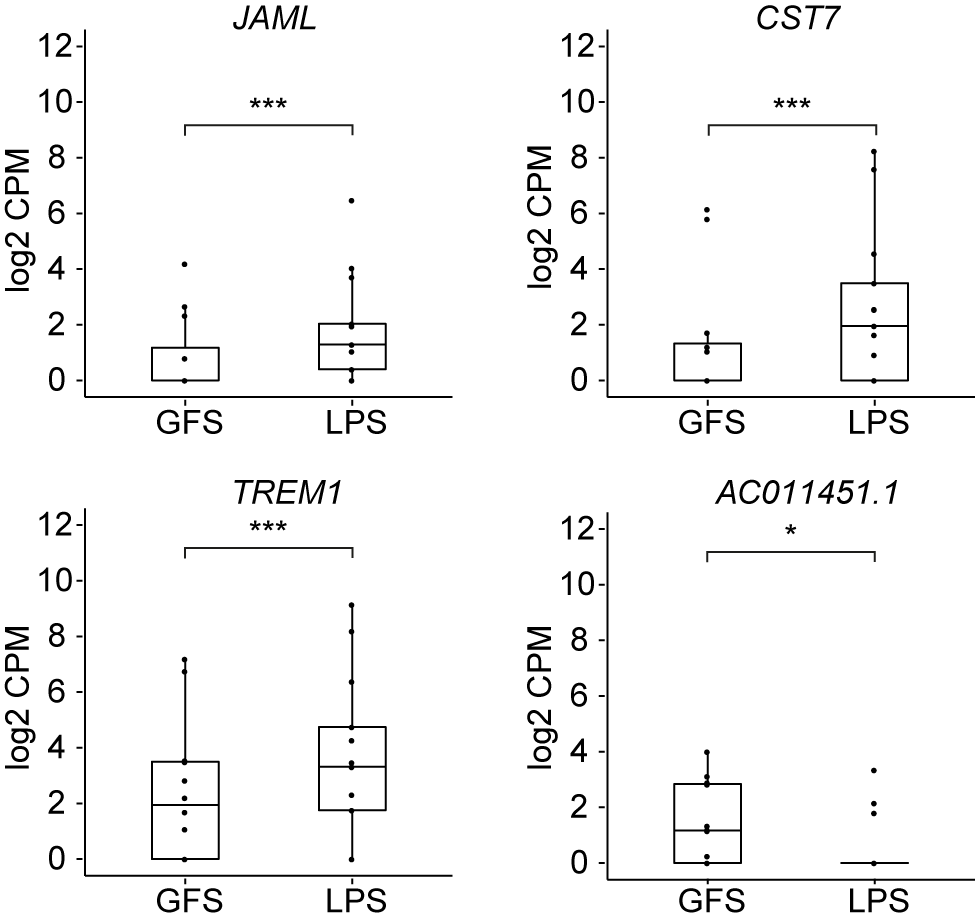

Supplement: FIGURE S3 — Differentially expressed genes between parietal (LPS) and frontal (GFS) brain regions. Selected examples of expression levels of genes differentially expressed between LPS and GFS shown in log2 CPM (likelihood ratio test; JAML ***FDR < 0.001, CST7 ***FDR < 0.001 and TREM1 ***FDR < 0.001, AC011451.1 * FDR = 0.04). Abbreviations: CPM, counts per million; GFS, superior frontal gyrus; LPS, superior parietal lobe. [file Image_3.TIF]

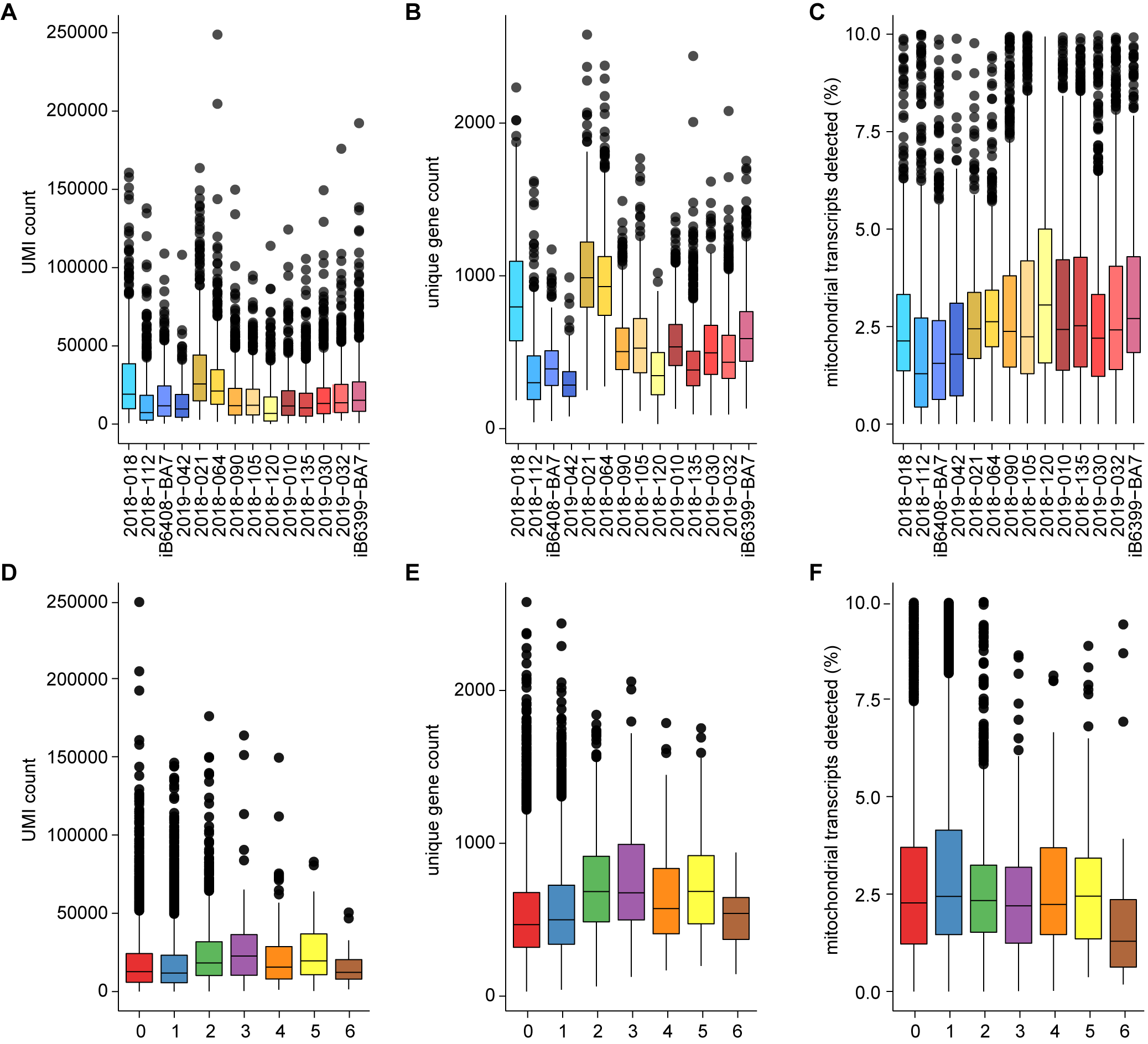

Supplement: FIGURE S4 — Bc-Smart-seq2 single-cell RNA sequencing quality of donors and clusters. (A–F) Boxplots displaying the number of detected UMIs, unique genes and percentage of mitochondrial transcripts in single cells per donor (A,B,D) and per cluster (D–F). Abbreviations: AD, clinically diagnosed Alzheimer’s Disease with Aβ plaques and/or hyperphosphorylated tau; CTR, Control; CTR+, Control with Aβ plaques and/or hyperphosphorylated tau, UMI; unique molecular identifier. [file Image_4.TIF]

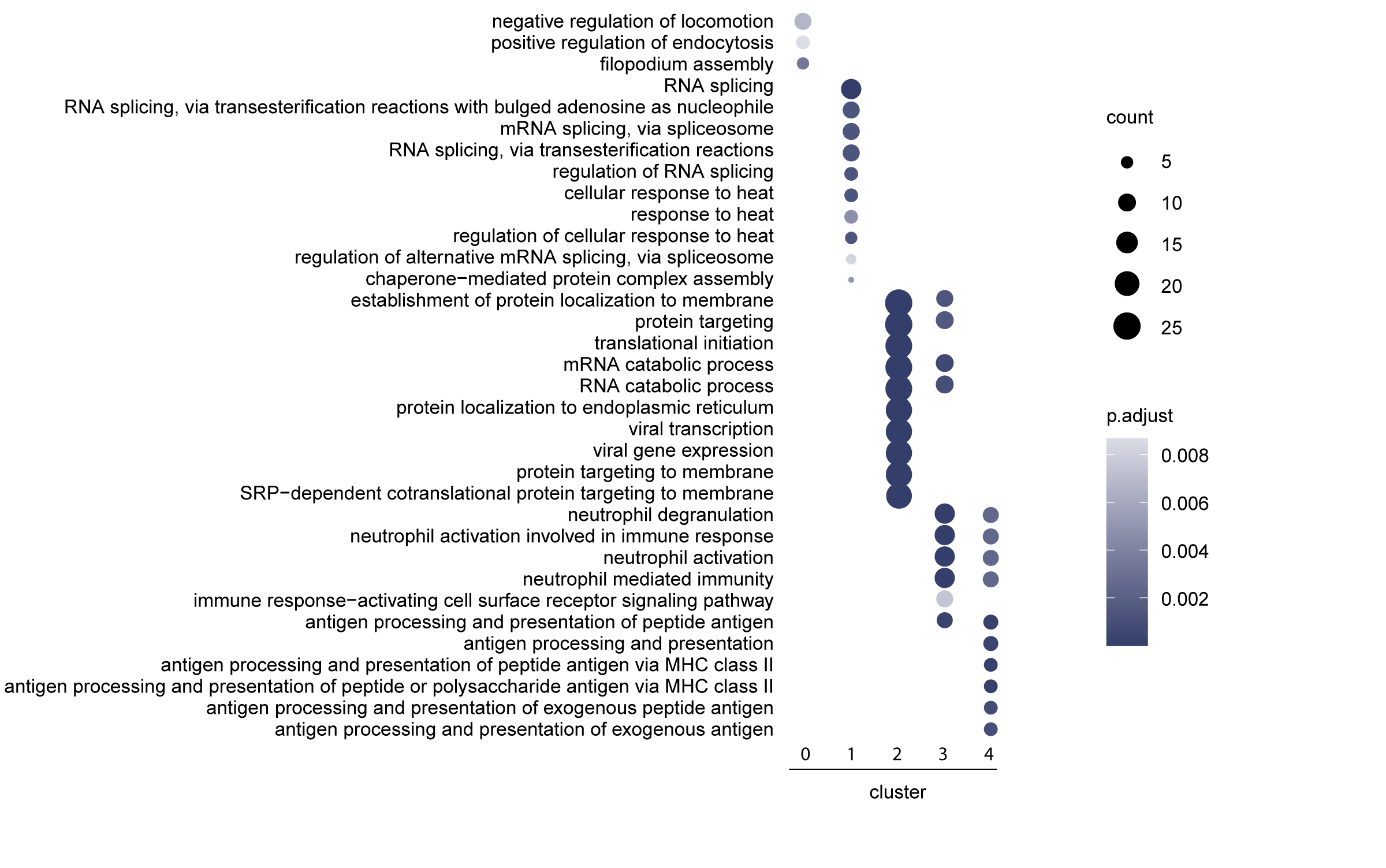

Supplement: FIGURE S5 — Biological annotation of marker genes for each cluster. Dot plots displaying the top 10 gene ontology terms per cluster identified in bc-Smart-seq2 single-cell data. Gene ontology terms were ordered on gene count. Color indicates the adjusted p-value. No GO terms were significantly associated with genes of clusters 5 and 6. [file Image_5.TIF]
